# Differential Effects of Genistein on Prostate Cancer Cells Depend on Mutational Status of the Androgen Receptor

**DOI:** 10.1371/journal.pone.0078479

**Published:** 2013-10-22

**Authors:** Abeer M. Mahmoud, Tian Zhu, Aijaz Parray, Hifzur R. Siddique, Wancai Yang, Mohammad Saleem, Maarten C. Bosland

**Affiliations:** 1 Department of Pathology, University of Illinois at Chicago, Chicago, Illinois, United States of America; 2 Center of Pharmaceutical Biotechnology, University of Illinois at Chicago, Chicago, Illinois, United States of America; 3 Section of Molecular Chemoprevention and Therapeutics, the Hormel Institute, University of Minnesota, Austin, Texas, United States of America; 4 Department of Pathology, Xinxiang Medical University, Xinxiang, China; Innsbruck Medical University, Austria

## Abstract

Blocking the androgen receptor (AR) activity is the main goal of therapies for advanced prostate cancer (PCa). However, relapse with a more aggressive, hormone refractory PCa arises, which harbors restored AR activity. One mechanism of such reactivation occurs through acquisition of AR mutations that enable its activation by various steroidal and non-steroidal structures. Thus, natural and chemical compounds that contribute to inappropriate (androgen-independent) activation of the AR become an area of intensive research. Here, we demonstrate that genistein, a soy phytoestrogen binds to both the wild and the Thr877Ala (T877A) mutant types of AR competitively with androgen, nevertheless, it exerts a pleiotropic effect on PCa cell proliferation and AR activity depending on the mutational status of the AR. Genistein inhibited, in a dose-dependent way, cell proliferation and AR nuclear localization and expression in LAPC-4 cells that have wild AR. However, in LNCaP cells that express the T877A mutant AR, genistein induced a biphasic effect where physiological doses (0.5-5 µmol/L) stimulated cell growth and increased AR expression and transcriptional activity, and higher doses induced inhibitory effects. Similar biphasic results were achieved in PC-3 cells transfected with AR mutants; T877A, W741C and H874Y. These findings suggest that genistein, at physiological concentrations, potentially act as an agonist and activate the mutant AR that can be present in advanced PCa after androgen ablation therapy.

## Introduction

Prostate Cancer (PCa) is the most common malignancy and the second leading cause of cancer death among men in United States [1]. In Asian populations, the incidence of PCa is lower compared to that in USA and European countries [2]. Most epidemiological studies have shown an association between dietary consumption of soy and reduced risk of PCa in Asians, for many of whom soy foods are a primary source of protein [3–5]. Meta-analyses of epidemiological studies support a protective effect of soy [5,6]. Dietary soy is rich in isoflavones, including the principal isoflavone compounds genistein and daidzein as well as less abundant compounds such as glycitein [7,8]. Genistein is the most abundant and biologically active isoflavone in soy. The steady state genistein concentrations in plasma of Japanese consuming soy-rich diets are as high as 2.4 µmol/L which is several fold higher than that of Europeans [9,10]. 

There is a growing body of evidence that genistein has anticarcinogenic effects on PCa [3,11]. It modulates the expression of some genes that control cell survival, cell cycle, and apoptosis [12], inhibits tyrosine kinase activity [13], and NF-κB [14], regulates the Akt and MAPK signaling pathways [15], and inhibits angiogenesis and metastasis [16–21]. Genistein also has antioxidant properties [22] and in some *in vitro* studies genistein reduced expression and transcriptional activity of the androgen receptor (AR) [23–29]. 

PCa is an androgen-dependent disease and various therapeutic modalities are directed toward androgen ablation for locally advanced or metastatic PCa. Most patients who receive androgen ablation therapies initially show clinical and biochemical response (decreased serum levels of prostate-specific antigen [PSA]). However, virtually all of those patients relapse with a more aggressive, hormone refractory (castration-resistant) form of PCa which does not require circulating androgen, but still depends on functional AR for growth and progression. There are several proposed mechanisms for the molecular switch of PCa from an androgen-dependent to an androgen-independent state, including evidence to suggest that the growth of most recurrent PCa is driven by inappropriate activation of the AR [30–32]. AR activity in the absence of testicular androgens can occur through several mechanisms, including AR amplification, deregulation of growth factors or cytokines, alteration of coactivators, and local production of androgens within the prostate [33–38]. Another mechanism is the acquisition of AR mutations that cause the receptor either to be hypersensitive to low concentrations of androgens or to expand its ligand specificity when they occur in the ligand binding domain (LBD) [39,40]. The latter types of mutations enable the receptor to be activated by a broad range of steroids such as estrogens, progestins, adrenal steroids, or even antiandrogens [41,42]. For example, a threonine to alanine mutation in the AR codon 877 (T877A) exist in up to 12.5% of hormone-refractory PCa and allows the AR to be activated by 17β-estradiol, progesterone, and some antiandrogens [42]. This inappropriate promiscuous binding of non-androgen ligands possibly contributes to treatment resistance in patients with advanced PCa [43]. 

Genistein has a 17β-estradiol-like structure and has estrogenic activity in breast cancer cells [44]. Although in a number of *in vitro* studies genistein downregulated AR transcription and PSA protein expression in PCa cells and inhibited their growth [24,26,27], stimulatory effects have been also reported [45–47]. However, most of these studies have some methodological limitations. First, pharmacological or even cytotoxic concentrations of genistein that do not reflect what can be achieved by humans consuming soy have been used in many of these studies. Second, in most studies examining the effects of genistein on PCa, LNCaP cells have been utilized. This cell line has a T877A mutation in the LBD of the AR that extends its binding specificity, enabling activation by other steroids or steroid like molecules [48]. We hypothesize that genistein with its estradiol-like structure could be a potential ligand for this mutant AR, which potentially explains the stimulatory effects that have been obtained using LNCaP cells in some studies. Nevertheless, there are no studies of the differential effects of genistein at physiological *versus* pharmacological doses on PCa cells with wild type ARs (WT-ARs) and cells with mutant ARs. In the current study, we employed two PCa cell lines; LAPC-4 cells that have WT-ARs [49] and LNCaP cells with T877A mutant ARs [48]. Cells were treated the cells with a wide range of genistein doses, including physiologically attainable concentrations, to determine the role of the T877A AR mutation and genistein concentration on genistein effects on cell proliferation, apoptosis, and AR and PSA expression. 

## Materials and Methods

### Chemicals and reagents

Genistein and Casodex were purchased from Sigma Aldrich (St. Louis, MO). Methyltrienolone (R1881) was purchased from Perkin Elmer (Downers Grove, IL).

### Culture of PCa Cell Lines

LAPC-4 cells were obtained from Dr. R. Reiter via Dr. Karen Knudsen (UCLA), which was originally developed by Dr. Charles Sawyers [49]. LNCaP and PC-3 cell lines were obtained from the ATCC (Rockville, MD). Cells were maintained in phenol red free RPMI media with L-glutamine supplemented with 10% FBS, 100 IU/mL Penicillin, and 100 μg/mL streptomycin. Media were replaced with RPMI containing 10% charcoal stripped fetal bovine serum 24 hours before treating cells with 1 μL/mL DMSO vehicle in which genistein was dissolved at concentrations of 0.5, 1, 5, 10, 25, and 50 μmol/L, with or without 100nmol/L Casodex (Sigma, St. Louis, MO), and/or 1 nmol/L methyltrienolone (R1881) (Perkin Elmer, Downers Grove, IL).

### Cell Proliferation Assay

Cell survival was assayed using the Cell Titer 96 AQ_ueous_ Non-Radioactive Cell Proliferation Assay (MTS) (Promega, Madison, WI). Cells were seeded in 96-well plates (3,000 cells/well). Twenty μL MTS reagent/100 μL medium were added and then left for 4 hours in the humid incubator at 37°C. The absorbance was recorded at 570 nm using microplate reader (Synergy 2, Biotek, Highland Park, VT). Cell proliferation and cell viability were also measured by hemocytometer counting; cells were plated onto 24-well cell culture plates at 5,000 cells/well in 1 ml of culture medium with FBS. At 24, 48, and 72 h after treatment, cells were harvested by trypsin solution. Cell counts were performed in triplicates using a hemocytometer with trypan blue (0.2%) exclusion to identify viable cells. The total numbers of viable and dye-stained cells in each experiment were compared with those of the corresponding untreated control cell counts performed simultaneously in three independent experiments.

### Cell cytotoxicity assay

Cell cytotoxicity was assayed using the Vybrant Cytotoxicity Assay Kit (G6PD release Assay) (Invitrogen, Grand Island, NY). Cells were seeded in 96-well plates (5,000 cells/well) in a 50 μL media. After 48 hours of treatment, 50 μL of the pre-prepared 2X resazurin/reaction mixture were added to each well and incubated at 37°C for 30 minutes. The fluorescence was recorded using fluorescence microplate reader that was set up with excitation and emission filters suitable for resorufin (excitation 530-560 nm, emission 580-600 nm).

### Transfection of PC-3 Cells

PC-3 cells, 5,000 cells/well, were transiently transfected on 24-well plates with 0.5 µg of WT-AR, T877A-AR, W741C-AR, H874Y-AR, or pSG5 empty vector using Lipofectamine^TM^ LTX (Invitrogen Grand Island, NY). Plasmids were generously provided by Dr. Karen Knudsen (Kimmel Cancer Center, Thomas Jefferson University, Philadelphia, PA). Eight hours after transfection, cells were treated with vehicle (ethanol and/or DMSO) or genistein. 

### Flow Cytometric Analysis

Cells were seeded into T-25 flasks at a concentration between 5 x 10^5^ - 10^6^ cells and treated with genistein for 48 hours. Treated cells were harvested, washed in cold phosphate-buffered saline (PBS) and stained for Annexin V to assess apoptosis using the Alexa Fluor 488 annexin V/Dead Cell Apoptosis Kit (Invitrogen). For cell cycle analysis, cells were fixed with 70% ethyl alcohol for at least 15 min and stained with propidium iodide solution (50 μg/mL propidium iodide, 0.1mg/mL RNase A, and 0.05% Triton X-100). Cells were incubated for 40 minutes at 37°C and then analyzed using the CyAn ADP three channel flow cytometer and Summit3 software (Beckman Coulter, Brea, CA). 

### Western Blot Analysis

Total protein was isolated from treated cells and for semiquantitative analysis of nuclear AR, nuclear and cytoplasmic extracts were isolated using a Nuclear Extract Kit (Active Motif, Carlsbad, CA). Protein concentration was measured at 595 nm with a microplate reader using the Bio-Rad Protein Assay kit (Bio-Rad Laboratories, Hercules, CA). Twenty five μg protein/lane was resolved by NuPAGE 4-12% Bis-Tris Gel (Invitrogen) and transferred to PVDF membranes. The immunoblot was incubated with primary antibodies at dilutions of 1:500-diluted rabbit polyclonal antibody for AR (N-20), 1:500 goat polyclonal antibody for PSA (C-19) (Santa Cruz Biotechnology, Santa Cruz, CA) or 1:1000 mouse monoclonal antibody for phosphotyrosine (4G10) (Invitrogen) overnight at 4°C. Secondary antibodies conjugated to horseradish peroxidase were used (Promega). ECL Western blotting detection reagents (Amersham Biosciences, Piscataway, NJ) were used to identify the antibody-bound protein bands. β-Tubulin and Tata Binding Protein (TBP) (Cell Signaling, Danvers, MA) were used as loading controls for total protein and nuclear protein, respectively. Image J software was employed to calculate the intensity of the AR and PSA bands relative to the housekeeping gene in three independent experiments.

### Real-Time PCR

Total RNA was extracted using the RNeasy mini kit (Qiagen, Germantown, MD). RNA quantities and qualities were determined by measuring absorbance at 260 and 280 nm by spectrophotometry. Five μg of total RNA were reversely transcribed into cDNA using SuperScript RT III (Invitrogen). AR and PSA mRNA expression were determined by real-time RT-PCR using the SYBR Green Assay standard protocol, and the step one plus Real-Time PCR System (Applied Biosystems, Foster City, CA) was used. Specific primers for each gene were designed. For AR, the forward primer was 5´-TTGTGTCAAAAGCGAAATGG- 3´, and the reverse primer was 5´- CAATGGGCAAAACATGGTC-3´. For PSA, the forward was 5´- CTTGTAGCCTCTCGTGGCAG-3´, and the reverse primer was 5´-GACCTTCATAGCATCCGTGAG- 3´. Glyceraldehyde 3- phosphate dehydrogenase (GAPDH) was used as the internal control to normalize expression data. GAPDH forward primer was 5´- GCCTCAAGATCATCAGCAATGCCT-3´, and the reverse primer was 5´- TGTGGTCATGAGTCCTTCCACGAT- 3´. Threshold cycle numbers (Ct) generated by the real-time RT-PCR were used to calculate the normalized expression ratio of the target genes using the 2^-∆∆Ct^ (Livak) method. Reactions were carried out in triplicates, and the results show three independent experiments.

### Luciferase Assay

Cells were seeded into 24 well-plates at a density of 5000 cells/well in standard medium without antibiotics. Cells were transiently co-transfected with PSA promoter-firefly luciferase plasmid and *Renilla* luciferase expression vector as a control for transfection efficiency using Lipofectamine 2000 (Invitrogen). Both plasmids were generously provided by Dr. Larisa Nonn (UIC, Chicago, IL). Eight hours after transfection, cells were treated with vehicle (ethanol and/or DMSO) or genistein. After 24 hours, the cells were harvested, and reporter and *Renilla* luciferase activities were determined using the Dual–Luciferase Assay System (Promega).

### Nuclear Localization Studies

Cells were plated in 16-well chamber slides (Lab-Tek chamber slide system, Nalge Nunc, Naperville, IL). Thirty minutes - 4 hours after treatment, cells were rinsed with 1x PBS, fixed with 4% formaldehyde/1xPBS for 20 minutes and permeabilized with 0.1% Triton X-100/1xPBS for 10 minutes. Cells were incubated first with 5% serum free protein block for 60 minutes and then with AR primary antibody (N-20 from Santa Cruz Biotechnology) at 1:200 dilution overnight at 4°C in a humidified chamber. The next day, cells were washed and incubated with the FITC-conjugated anti-rabbit secondary antibody (Santa Cruz Biotechnology) for 1 hour then incubated with 1:500 Hoechst counterstaining for 20 minutes in the dark. Slides were examined under inverted fluorescent microscope (Eclipse TE 2000, Nikon, Japan) equipped with a cooled CCD camera (Photometric Cascade II, Tucson, AZ) under control of MetaMorph imaging software (Molecular Devices, Fully Vale, CA). 

### ^3^[H]-R1881Competitive Binding Assay

This analysis was done by using the method described by Siddique et al. [50]. Briefly, LAPC4 and LNCaP cells were seeded in 12-well plates in phenol red-free media containing 5% charcoal stripped FBS for 3 days after which, media were replaced with serum-free media containing 1nM ^3^[H]-R1881 with or without 0.1–1,000-fold molar excess of unlabeled competitor ligands (R1881, Casodex or genistein) for 90 min at 37°C. Cells were washed with phosphate buffer, and the bound ^3^[H]-R1881 was extracted in ethanol for 30 min at room temperature and detected by scintillation counting. 

### Genistein Binding with AR in silico

The X-ray crystal structure of wild-type androgen receptor (PDB code 1I37) and T877A mutant (PDB code 1I38) in combination with dihydrotestosterone (DHT) were prepared via the Protein Preparation Wizard of the Schrodinger Suite 2011. Genistein construction was prepared using LigPrep [51]. OPLS2005 force field was used for geometric optimization, and possible ionization and tautomeric forms were created for genistein at pH 7.5 ± 0.5 using EPIK [52]. Molecular docking was performed with GOLD v5.0.1 [53]. The binding site sphere was defined with a 10 Å radius around the ligand present in the crystal structure, and 100 docking runs were performed for each ligand. Default values were used for other genetic algorithm parameters. All calculations were performed with the Amber 11 suite of programs [http://ambermd.org/], including the ff99SB force field for the AR protein, the GAFF force field for the ligand, and MM/PBSA to calculate binding free energies [54]. Each protein-ligand complex was solvated in an octahedral box of TIP3P water molecules extending 10 A° outside the protein on all sides. The electrostatics were treated with the particle-mesh Ewald method [55]. The simulations used a residue-based cutoff of 8 Å, a time step of 2 fs and a constraint of bond lengths involving hydrogen atoms using the SHAKE algorithm. The solvated complexes were minimized with 10000 steps of conjugate gradient minimization, equilibrated with MD at 300 K with 50ps of heating and 50 ps of density equilibration with 2 kcal mol^-1^ Å^-2^ restraints on the complex and followed by 500ps of constant pressure equilibration with 0.5 kcal mol^-1^ Å^-2^ restraints on the complex at 300K. After equilibration, 20 ns production run was performed to assess free energy convergence and coordinates were extracted every 2 ps. Root mean squared deviation (RMSD) analyses were performed using the ptraj module of Amber 11. MM/PBSA is a well-established method to calculate binding free energies [56]. The binding free energy was calculated using a simple thermodynamic cycle from the energy difference between the complex and the unbound forms. The values of the free energy of binding of each compound were calculated according to the equation Δ*G*_bind_ = *G*_complex_ - *G*_receptor_ - *G*_ligand_. The free energy of each of these was estimated as a sum of the four terms *G* = *E*_MM_ + *G*_psolv_ + *G*_npsolv_ - *TS*_lnmode,_ where E_MM_ is the molecular mechanics energy of the molecule expressed as the sum of the internal energy of the molecule plus the electrostatics and van der Waals interactions, Gpsolv is the polar contribution to the solvation energy of the molecule, Gnpsolv is the nonpolar solvation energy, T is the absolute temperature, and S is the entropy of the molecule estimated by normal mode analysis. The snapshots for MM/PBSA were taken every 20 ps of the last 2 ns MD production runs, resulting in a total of one hundred snapshots per run. Normal-mode analysis was employed to calculate the vibrational, rotational, and translational entropy. Because of the high computational cost and the deviation of entropy which is relatively small for different conformations [57], we only selected 12 regularly spaced snapshots along the last 2 ns production trajectory for entropy calculations. 

### Statistical Analysis

The data were analyzed using Student's t -test, or one-way ANOVA followed by a post-hoc test as appropriate and p values of <0.05 were considered significant. 

## Results

### Physiological Concentrations of Genistein Inhibited LAPC-4 Cell Growth, but Stimulated Growth of LNCaP Cells

To determine whether the T877A mutation of the AR influences the effects of genistein on PCa cell proliferation, we applied two approaches, (a) using cell lines that naturally have WT AR (LAPC-4 cells) or T877A mutant AR (LNCaP cells) and (b) using an AR-null cell line (PC-3) transfected transiently with exogenous WT or T877A mutant AR in order to overcome any biological discrepancies from using two different cell lines. Also, in order to avoid variation in levels of AR expression between PC-3 cells transfected with different AR constructs and LNCaP cells, a range of DNA concentrations (0.5-5µg) was tested and the optimal dose that led to a final AR expression level close to that in LNCaP cells was used (0.5µg). 

**Figure 1:**
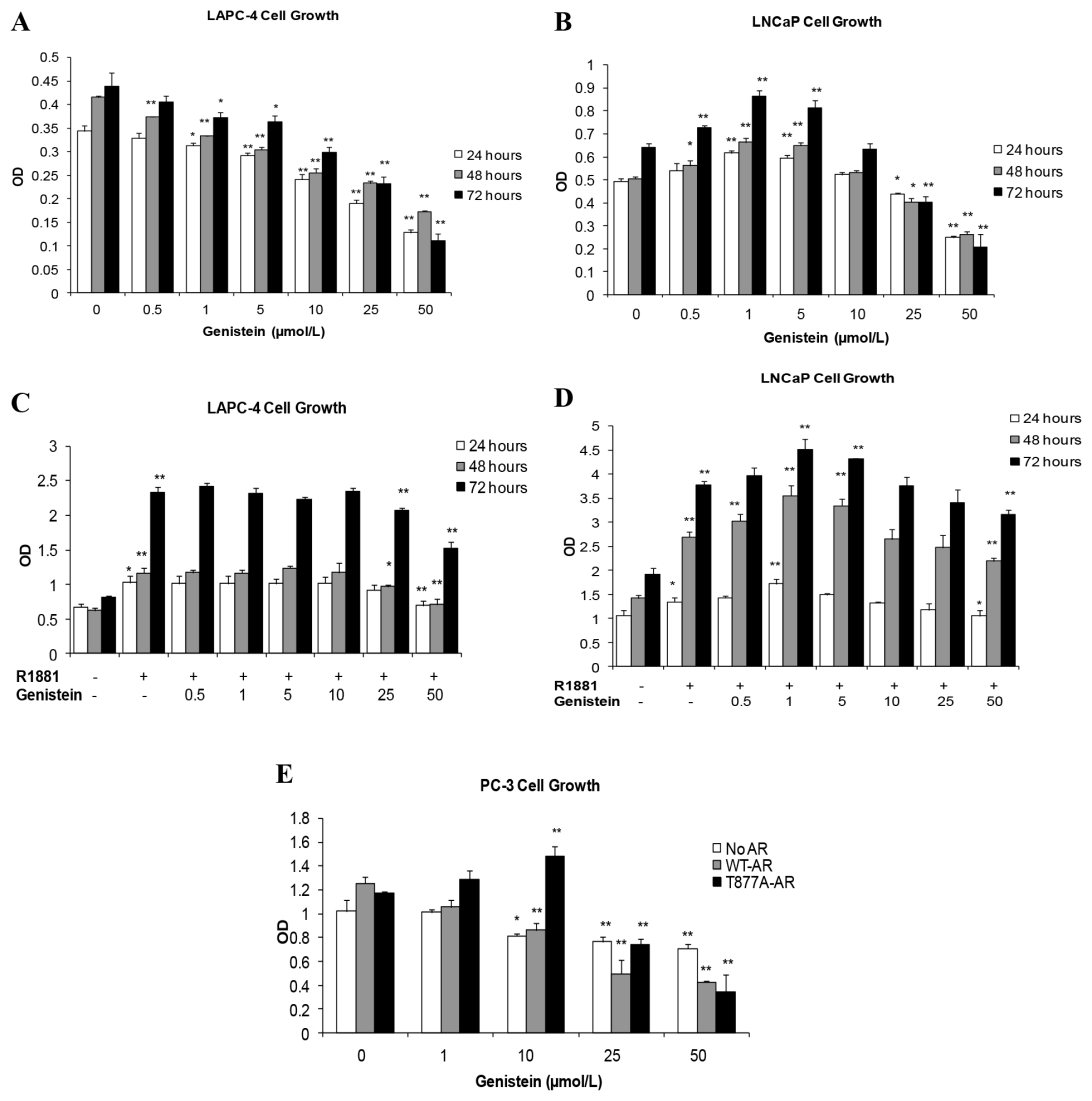
Effects of genistein on PCa cell growth and viability measured by MTS assay. The data represent the mean ± SD (standard deviation) of three experiments each in triplicate. A and B: Graphic presentation of the effects of different concentrations of genistein on LAPC-4 (A) and LNCaP (B) cell growth. C and D: The effect of combined treatment with genistein and R1881 on LAPC-4 (C) and LNCaP (D) cell proliferation. E: The effect of genistein treatment for 24 hours on the growth of non transfected PC-3 cells or PC-3 cells transfected with either WT-AR or the T877A mutant AR. OD; optical density. *p < 0.05, **p <0.01 for comparisons with the control groups.

PCa cells were treated with a range of genistein concentrations (0.5-50 µmol/L) for 24 to 72 hours, after which cell growth and viability were evaluated by both an MTS cell proliferation assay and hemocytometer cell counting with trypan blue exclusion. Genistein inhibited LAPC-4 cell proliferation significantly at all concentrations tested in a dose-related fashion (Figure 1A). In contrast, physiological concentrations of 0.5, 1.0, and 5.0 µmol/L genistein stimulated LNCaP cell proliferation by 13%, 35%, and 27% over controls, respectively, whereas higher concentrations of genistein (25 and 50 µmol/L) caused inhibition of proliferation by 38% and 50% (Figure 1B). In LAPC-4 cells, treatment with 1nmol/L R1881 abolished the inhibitory effects of genistein, except at a high concentration (50µmol/L) (Figure 1C). While in LNCaP cells, combined treatment with low dose genistein (1 µmol/L) and synthetic androgen (1 nmol/L R1881) additively increased cell proliferation by 134%, which was more than that caused by either genistein or androgen alone (35% and 90% relative to control, respectively) (Figure 1D). Hemocytometer counting with trypan blue dye exclusion showed a comparable pattern to that obtained by the MTS proliferation assay and a lack of cytotoxicity (data not shown). 

At 10 µmol/L or more, genistein treatment for 48 hours induced a significant inhibition of proliferation of PC-3 cells lacking AR expression (Figure 1E). Interestingly, the same concentrations of genistein induced a significant stimulation of proliferation of PC-3 cells expressing the T877A-mutant AR (Figure 1E), consistent with our findings in LNCaP cells that endogenously express the same mutant AR. Proliferation of PC-3 cells transfected with WT-AR was significantly inhibited at concentrations of 10 µmol/L or more (Figure 1E). 

In summary (see Table 1), PCa cells that express WT-AR either endogenously (LAPC-4 cells) or exogenously (WT-AR transfected PC-3 cells) did not exhibit any growth stimulatory response to genistein. However, PCa cells that express the mutant AR, either endogenously (LNCaP cells) or exogenously (transfected PC-3 cells), showed a significant enhancement in cell proliferation in response to lower micromolar concentrations of genistein.

**Table 1:** Summary of the overall effects of genistein on LAPC-4 and LNCaP cells.

**Genistein (µmol/L)**	**Cell Growth**	**Apoptosis**	**AR nuclear localization**	**AR protein and mRNA expression**	**PSA protein and mRNA expression**	**PSA luciferase activity**
**LAPC-4**						
0.5	↓	**↑**	**↓**	**↓**	**↓**	**↓**
1	↓	**↑**	↓↓	**↓↓**	**↓↓**	**↓↓**
5	↓↓	**N/A**	N/A	**↓↓↓**	**↓↓↓**	**↓↓**
10	**↓↓**	**↑↑**	**↓↓**	**↓↓↓**	**↓↓↓**	**↓↓↓**
25	**↓↓↓**	**↑↑↑**	**↓↓↓**	**↓↓↓**	**↓↓↓**	**↓↓↓**
50	**↓↓↓**	**↑↑↑**	**↓↓↓**	**↓↓↓**	**↓↓↓**	**↓↓↓**
**LNCaP**						
0.5	↑	↔	↑	↑	↑	↑
1	**↑↑**	**↔**	**↑↑**	**↑↑↑**	**↑↑↑**	**↑↑↑**
5	**↑↑**	**N/A**	**N/A**	**↑**	**↑**	**↑**
10	**↔**	**↔**	**↔**	**↔**	↓	**↓↓**
25	**↓↓**	**↑↑**	**↓↓**	**↓↓**	**↓↓↓**	**↓↓↓**
50	**↓↓↓**	**↑↑↑**	**↓↓↓**	**↓↓↓**	**↓↓↓**	**↓↓↓**

Abbreviations are as follows: ↑ slightly increased; ↑ ↑ moderately increased; ↑ ↑ ↑ markedly increased; ↔ no change, ↓ slightly inhibited; ↓ ↓ moderately inhibited; ↓ ↓ ↓ markedly inhibited.

### Low Doses of Genistein Induced Apoptosis and Cell Cycle Arrest in LAPC-4 Cells, but not in LNCaP Cells

**Figure 2:**
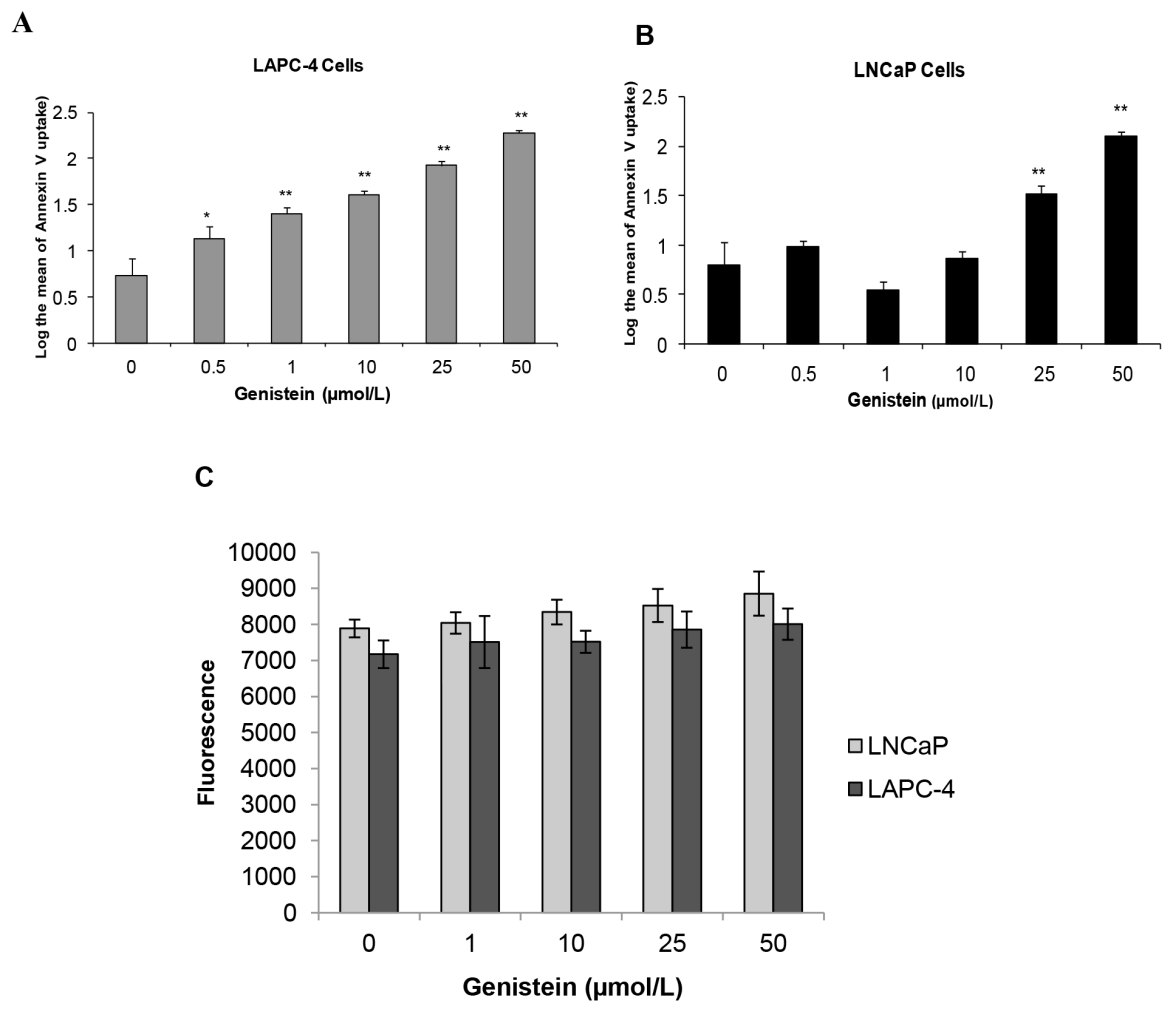
Effects of genistein on PCa cell apoptosis and cytotoxicity. Cells were treated with genistein (0.5- 50 µmol/L) for 48 h, then apoptosis was examined in LAPC-4 (A) and LNCaP cells (B) by flow cytometry after staining with Annexin-V apoptosis detection kit. Cells treated with genistein for 72 hours were tested for signs of cytotoxicity using the lactate dehydrogenase release assay. Results in the graph represent the mean ± SD of three experiments. *p < 0.05, **p <0.01, for comparisons with the control groups.

We compared LAPC-4 and LNCaP cells for the effects of increasing concentrations of genistein treatment for 48 hours on apoptosis and cell cycle progression, both assessed by flow cytometry. Apoptosis was augmented in a concentration-dependent manner in LAPC-4 cells, starting from a dose as low as 0.5 µmol/L (Figures 2A). In contrast, genistein concentrations between 0.5 and 10 µmol/L caused no significant change in apoptosis in LNCaP cells; apoptosis was increased only at genistein concentrations of 25 µmol/L or higher (Figures 2B). Consistent with these findings, genistein caused an increase of LAPC-4 cells in sub-G1 at concentrations equal to or higher than 10 µmol/L, whereas this was observed in LNCaP cells only at or above 25 µmol/L (Table 2).

**Table 2:** The effects of genistein on cell cycle distribution of LAPC-4 and LNCaP cells.

	LAPC-4 cells				LNCaP cells			
Genistein dose	Sub G	G0/G1	S	G2/M	Sub G	G0/G1	S	G2/M
Control	9.46 (3.8)	71.98 (5.1)	6.38 (3.3)	12.25 (2.8)	7.06 (1.5)	73.85 (2.5)	8.04 (2.1)	11.05 (2.8)
0.5 µmol/L	13.63 (1.5)	62.69 (9.4)	6.12 (4.9)	17.66 (2.6)	7.00 (1.5)	74.96 (5.4)	8.02 (2.9)	10.02 (4.6)
1.0 µmol/L	18.37 (6.2)	59.58*(4.1)	4.18 (0.8)	17.78 (5.6)	4.44* (0.9)	82.12* (4.3)	9.09 (2.4)	4.42* (2.4)
10 µmol/L	25.1**(2.6)	52.3**(2.9)	3.30 (0.9)	19.31*(2.8)	7.08 (1.5)	72.81 (4.8)	7.80 (1.8)	12.31 (2.6)
25 µmol/L	34.2**(4.2)	40.8**(4.3)	3.84 (4.1)	21.19*(3.5)	13.0** (2.5)	61.78**(3.2)	5.84 (2.1)	19.4* (3.6)
50 µmol/L	40.4**(0.8)	31.2**(3.0)	2.30 (0.9)	26.1**(2.0)	9.37** (1.6)	43.03**(3.4)	3.30*(1.1)	34.3** (3.9)

Propidium iodide-stained cells were analyzed using a flow cytometer. Results in the table represent the mean ± SD of three experiments.

* p<0.05, ** p<0.01, compared to the vehicle control group. Numbers between brackets are for standard deviations.

Regarding cell cycle progression, 1µmol/L of genistein significantly reduced LNCaP cells in sub-G1 and G2/M and increased cells in the G0/G1 phase of the cell cycle (Table 2). In contrast, in LAPC-4 cells, low doses of genistein induced growth arrest evident by reduction of the number of cells in G0/G1 and accumulation of cells in G2/M which is characteristic of an S-phase block or S/G^2^-phase transition block (Table 2). However, at supraphysiological concentrations (25µmol/L and higher), genistein causes a G2/M arrest and apoptosis in both cell lines, LAPC-4 cells being more sensitive than LNCaP cells. These findings are consistent with what we reported above in the MTS cell proliferation assay. To eliminate the effect of direct cytotoxicity of genistein, we then performed the lactate dehydrogenase release assay in both LAPC-4 and LNCaP cells across the whole range of genistein doses that have been used. There was no detectable cytotoxicity to any dose of genistein up to 50 µmol/L for 72 hours in either cell lines (Figure 2C). 

### The Stimulatory Effect of Genistein on LNCaP Cells was Mediated by the Mutant AR

**Figure 3:**
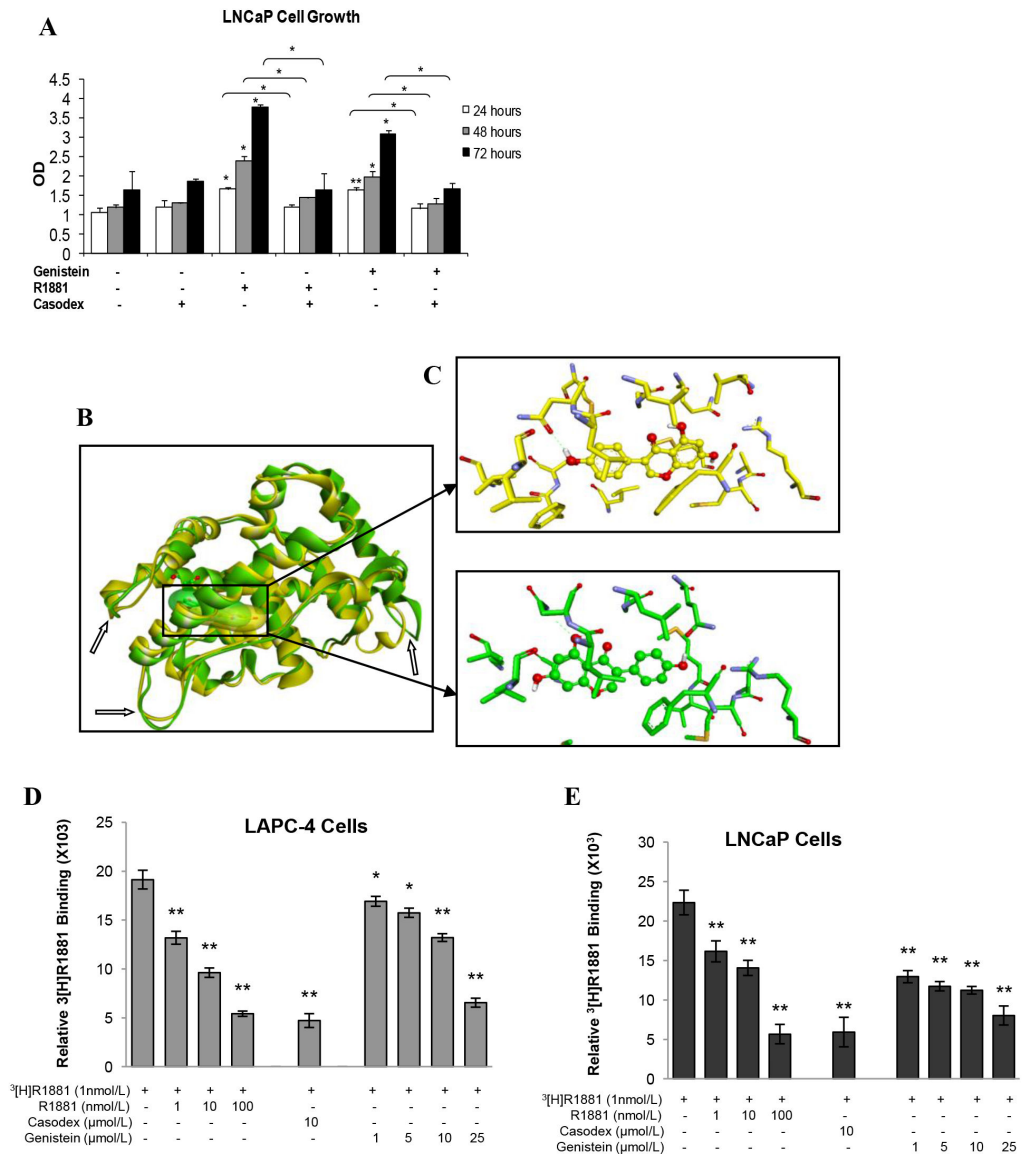
T877A mutant AR mediates the stimulatory effects of genistein. A: Graphic presentation of the effect of combined treatment with R1881 (1nmol/L) and Casodex (100nmol/L) or genistein (1µmol/L) and Casodex (100nmol/L) on LNCaP cell growth measured by MTS assay. Results in the graph represent the mean ± SD of three experiments each in triplicate. *p < 0.05, **p <0.01, for comparisons with the groups treated with R1881 or genistein, only. B: Evaluation of genistein–AR binding *in* *silico*. Representative figure showing conformational changes of the WT-AR and the T877A mutant AR after molecular docking with genistein *in* *silico* using the GOLD v5.0.1 modeling program. Different domains of the AR are presented in various colors. Inset, genistein bound to the ligand-binding domain (LBD) of AR-receptor. Arrows point to different conformational changes and loop movements in WT-AR and T877A mutant AR in response to genistein docking. C: Geometric optimization, and tautomeric forms of genistein created at pH 7.5 ± 0.5 using EPIK. The yellow is for the WT-AR, and the green is for the T877A mutant AR. D and E: Evaluation of genistein–AR binding using ^3^[R1881] competitive binding assay. Histogram shows the binding of genistein with AR in LAPC4 (WT-AR) and LNCaP (mutant AR) cells as assessed by radiolabeled cell-based competitive binding assay as described in Materials and Methods. Results in the graph represent the mean ± SD of three experiments. *p < 0.05, **p <0.01, compared with control.

We examined whether the mutant AR-T877A is required for genistein to enhance LNCaP cell proliferation in the absence of androgen (Figure 3A). LNCaP cells were incubated with either 1 µmol genistein or 1 nmol/L R1881 in the absence or presence of 100nM of the AR antagonist, Casodex. As shown in Figure 3A, Casodex alone did not affect LNCaP cell growth, but it abolished the stimulatory effect of 1 nmol/L R1881 on cell proliferation, consistent with the concept that AR activation mediates PCa cell proliferation (36,38). Adding 1µmol/L of genistein to the media enhanced LNCaP cell proliferation by 49% and this effect was also eliminated by 100nM Casodex. These findings indicate that the stimulatory effect of genistein on LNCaP cell proliferation is mediated primarily by the T877A mutant AR. 

The ability of genistein to enhance the proliferation in LNCaP cells, but not in LAPC-4 cells, may be due to different affinity by which genistein binds to the mutant AR and the WT-AR. To investigate this assumption, two approaches were employed, *in silico* molecular modeling and a ^3^[H]-R1881 competitive binding assay. *In silico* modeling showed that genistein binds to the AR binding pocket without overlapping with the DHT binding site (Figures 3B and 3C). The binding energies predicted by MM/PBSA for genistein binding to the WT-AR and the T877A mutant AR were -5.8 kcal/mol and -10.3 kcal/mol, respectively. These data suggest that genistein can bind to both the T877A mutant and the wild type forms of the AR, but with a two-fold higher binding affinity for the mutant AR than for the WT-AR, which provides a plausible explanation for the differential impact of genistein on cells with different mutational AR status. 

To probe the potential of genistein to compete with binding of the synthetic androgen (R1881) to the AR, LAPC4 and LNCaP cells were incubated with 1nmol/L of the ^3^[H] -R1881 and genistein. Genistein displaced the ^3^[H]-R1881 significantly in both LNCaP and LAPC-4 cells (Figures 3D and 3E) albeit with much higher affinity in LNCaP than LAPC-4 cells, which is more noticeable at low physiological concentrations (≤10µmol/L). Genistein, at 1µmol/L dramatically inhibited (42%) the binding of ^3^[H]-R1881 to the promiscuously mutant AR receptor in LNCaP cells compared to just 11.5% inhibition in LAPC-4 cells. However, at higher concentrations (>25 µmol/L) the competitive binding capacity of genistein to the AR was quite similar in both cell lines (data not shown). These findings suggest that genistein binds competitively to both types of AR, but they do not justify the possible mode of binding of genistein to WT-AR and T877A mutant AR (whether agonistic or antagonistic mode). Given that the T877A mutation proved able to modify the response of AR to many steroids and steroid like structures such as estrogen, progesterone, bisphenol A, and even the anti-androgens such as flutamide, it could be postulated that the presence of this mutation in the AR may change the mode and eventually the function of genistein binding to the mutant AR compared to the wild AR. Thus, we sought to evaluate the functional consequences of genistein binding to both types of AR via investigating the AR nuclear localization, expression and transcriptional activity after treatment with genistein in both LAPC-4 (WT-AR) and LNCaP (mutant AR) cell lines.

### Genistein at Low Concentrations Reduced AR Nuclear Localization in LAPC-4 Cells and Increased AR Nuclear Localization in LNCaP Cells

**Figure 4:**
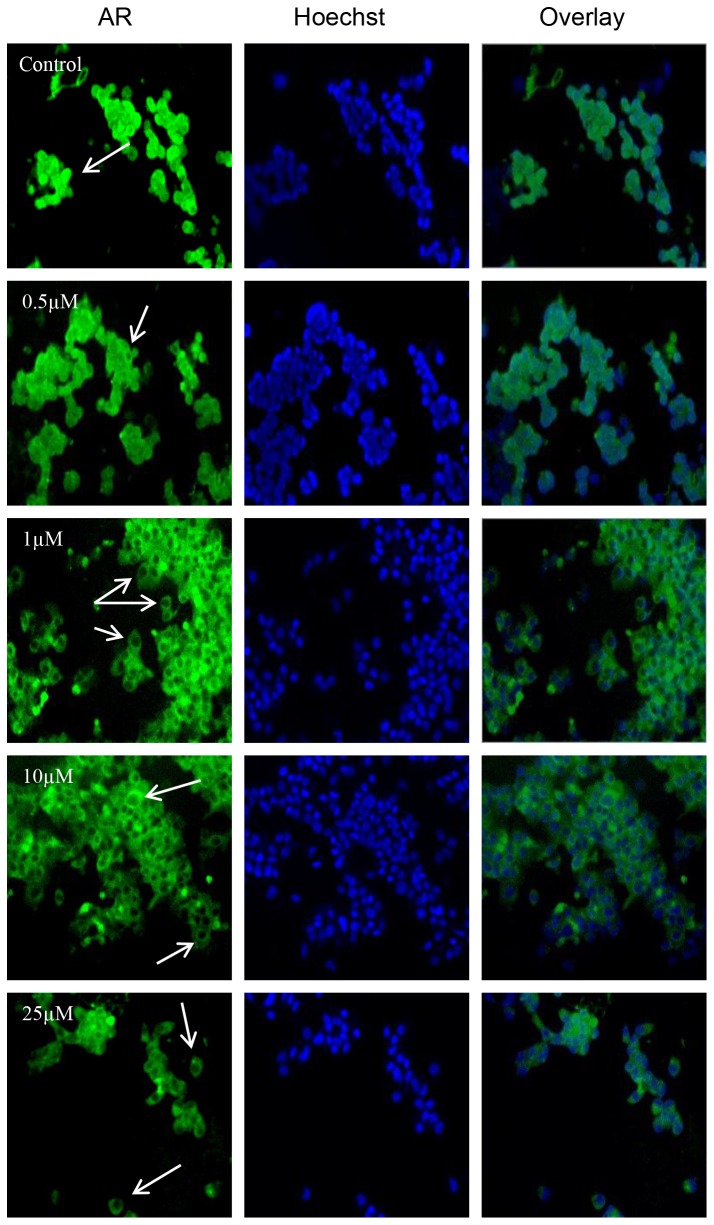
Effects of genistein on AR nuclear localization in LAPC-4 cells. Cells were treated with DMSO or genistein 0.5 µmol/L, 1µmol/L, 10µmol/L, and 25 µmol/L for 4hr. LAPC-4 cells were fixed and immunostained with anti-AR antibody. The green fluorescent staining is for AR (left column), the blue is for Hoechst counter staining (middle column) and the right column is the overlay for both staining.

**Figure 5:**
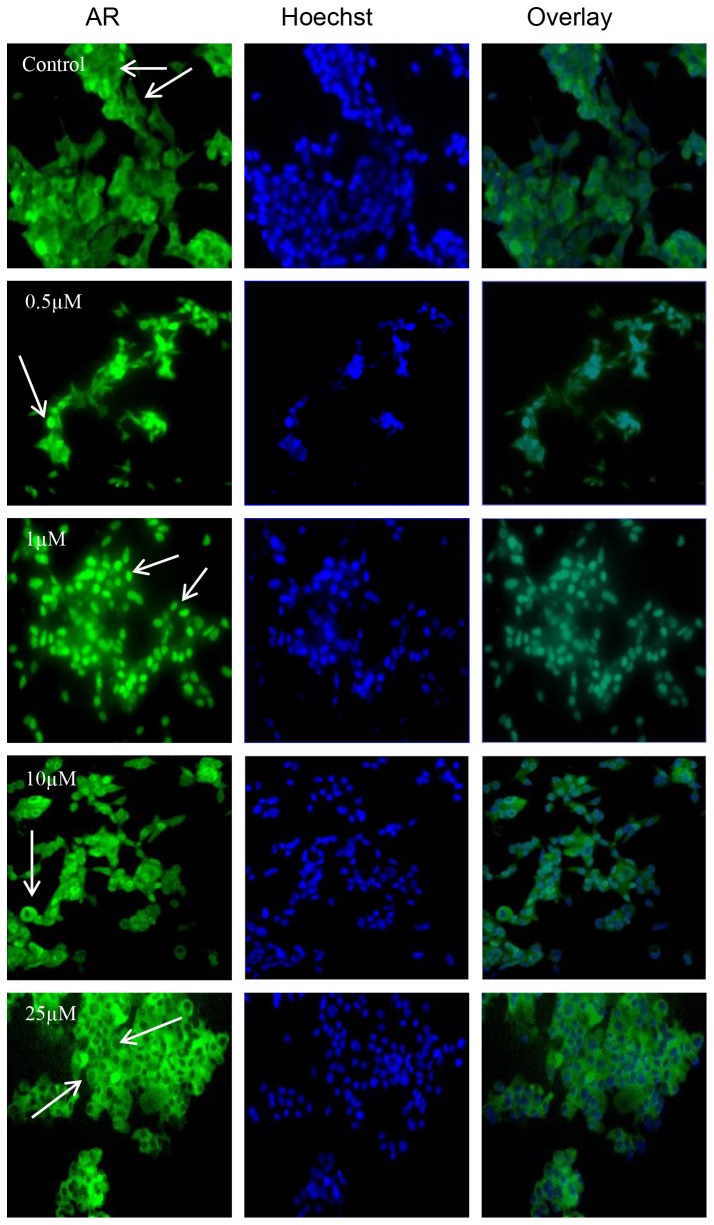
Effects of genistein on AR nuclear localization in LNCaP cells. Cells were treated with DMSO or genistein 0.5 µmol/L, 1µmol/L, 10µmol/L, and 25 µmol/L for 4hr. LNCaP cells were fixed and immunostained with anti-AR antibody. The green fluorescent staining is for AR (left column), the blue is for Hoechst counter staining (middle column) and the right column is the overlay for both staining.

**Figure 6:**
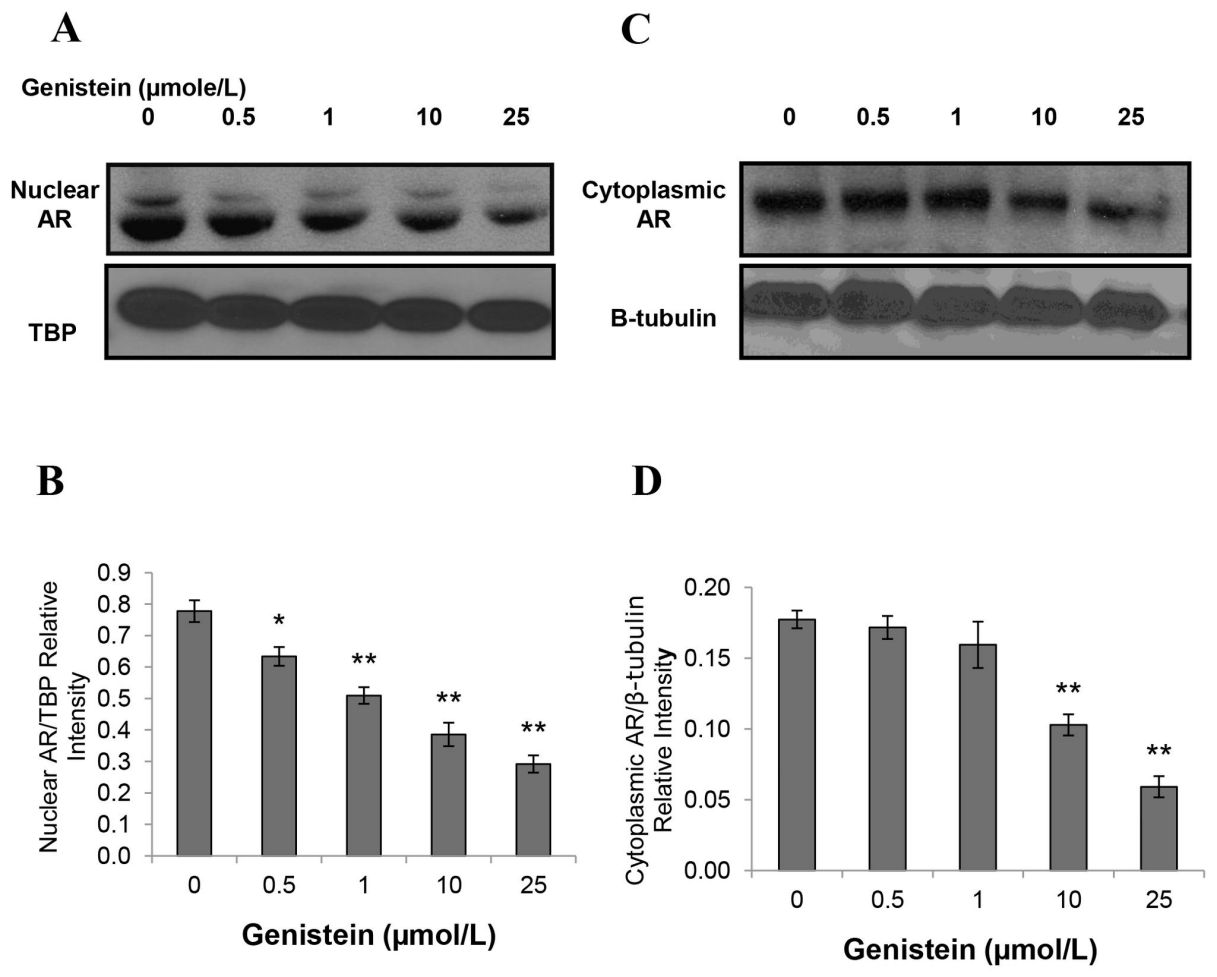
Effects of genistein on phosphorylated nuclear fraction of AR in LAPC-4 cells. Western blot analysis of the effect of different concentrations of genistein on the nuclear phosphorylated (A) and cytoplasmic fractions (C) of the AR protein in LAPC-4 cells. B and D show quantitative analysis of the signal normalized to TATA Binding Protein (TBP) and β-tubulin, respectively.

**Figure 7:**
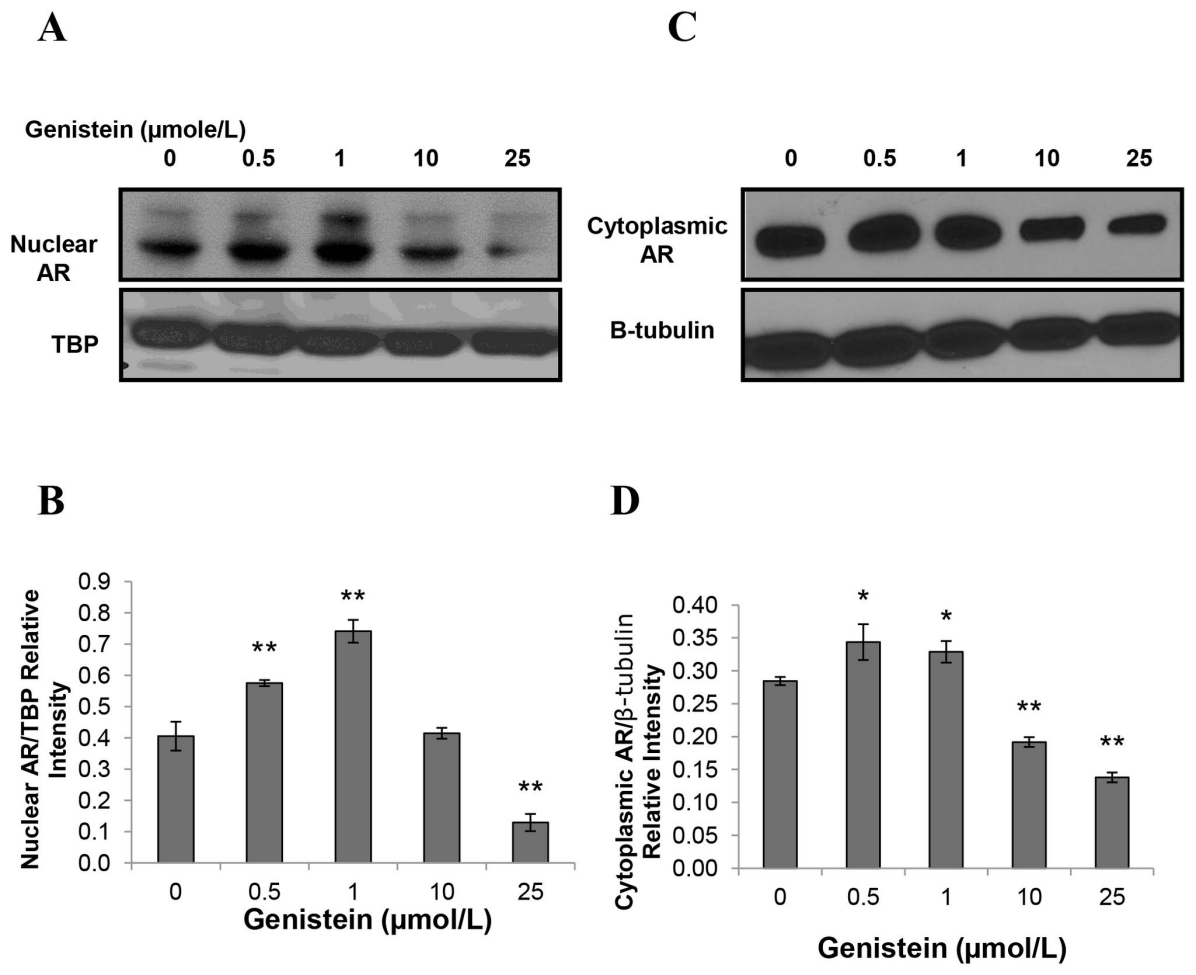
Effects of genistein on phosphorylated nuclear fraction of AR in LNCaP cells. Western blot analysis of the effect of different concentrations of genistein on the nuclear phosphorylated (A) and cytoplasmic fractions (C) of the AR protein in LNCaP cells. B and D show quantitative analysis of the signal normalized to TATA Binding Protein (TBP) and β-tubulin, respectively.

Because AR translocates rapidly to the nucleus upon activation [58], we compared the genistein effects on activation or subcellular localization of the AR in LAPC-4 and LNCaP cells. For these experiments, both cell lines were treated with either 1 nmol/L R1881 or genistein (0.5 to 50 µmol/L) in steroid free media for 30 minutes to 4 hours. Subcellular localization of AR was detected via two different methods, immunofluorescence and immune blotting of the nuclear fraction of cellular proteins. The immunoblot was labeled with the rabbit polyclonal anti-AR. The immunofluorescence analysis indicated that both LAPC-4 and LNCaP cells displayed a significant increase in AR nuclear localization in response to 1nmol/L R1881 after 30 minutes of exposure (data not shown). Genistein in steroid free media caused a reduction of AR nuclear localization in LAPC-4 cells in a dose dependent manner after 4 hours (Figure 4). In contrast, in LNCaP cells lower dosages of genistein (<10µmol/L) caused increased AR nuclear localization and only higher dosages (≥ 10µmol/L) of genistein reduced AR amount and nuclear localization (Figure 5). Phosphorylated AR assayed by immunoblot in nuclear extracts of LAPC-4 cells was significantly reduced in response to all doses of genistein starting from the lowest dose (0.5µmol/L) (Figures 6A and 6B). However, the cytoplasmic fraction of AR did not show any significant changes in response to these low physiological doses and a significant reduction was achieved only at higher dosages (≥ 10µmol/L) (Figures 6C and 6D). These findings indicate that genistein binding to the WT-AR in LAPC-4 cells is adjunct to a significant inhibition of AR nuclear localization. In LNCaP cells, in contrast, the phosphorylated AR was substantially increased in response to lower doses of genistein (0.5 and 1 µmol/L) (Figures 7A and 7B). This increase in the nuclear AR was accompanied by a parallel increase in the cytoplasmic AR (Figures 7C and 7D) which may imply that the effect of genistein on the AR extends beyond modifying its nuclear localization to include its level of expression too, which is addressed below. Collectively, these data demonstrate variable outcomes of genistein binding to the AR between cells with WT-AR (LAPC-4) and cells with promiscuously mutant AR (LNCaP). 

### Impact of Genistein on PCa Cell Growth and AR Nuclear Localization was Associated with Alterations in AR mRNA and Protein Expression

**Figure 8:**
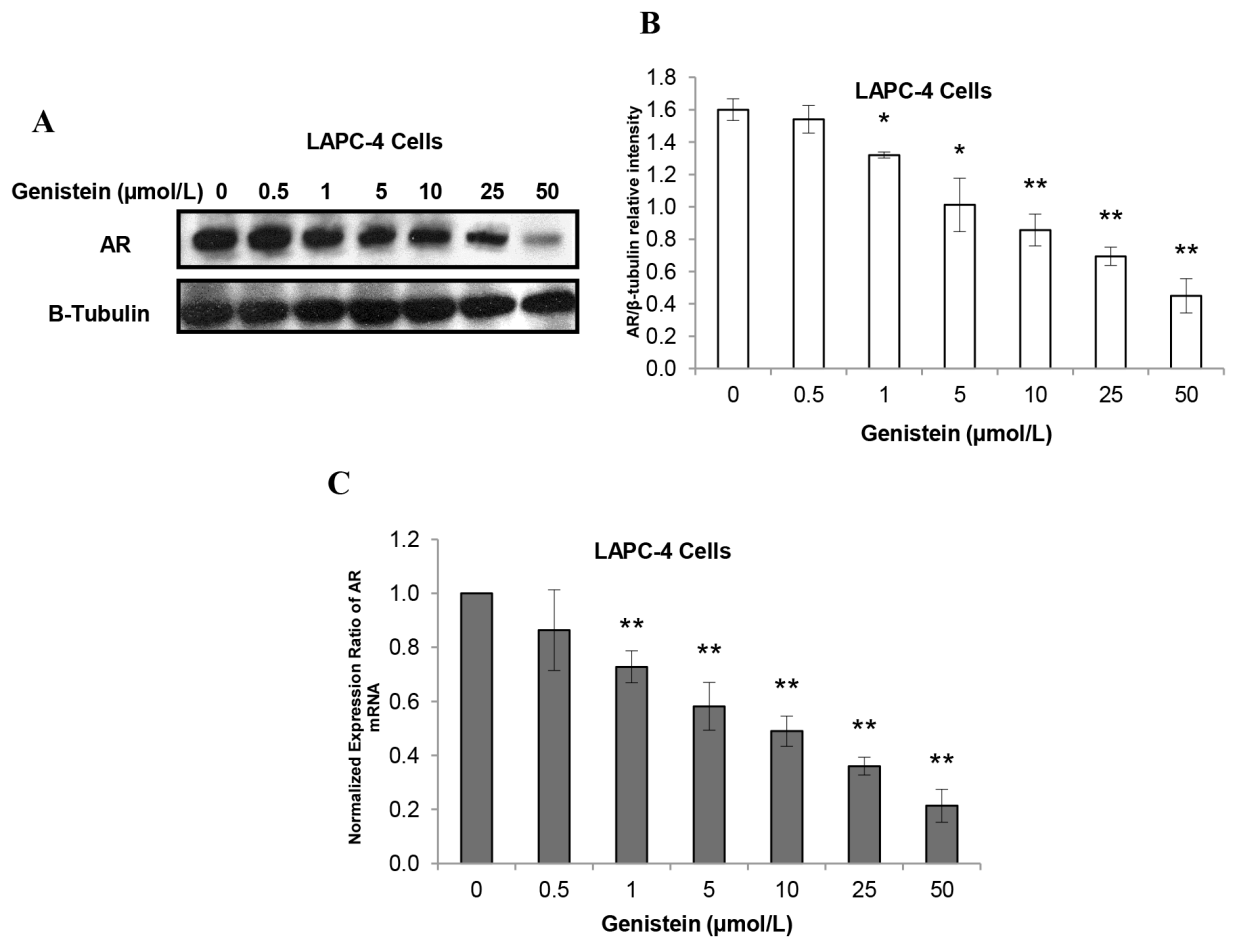
Genistein-mediated effects on AR protein and mRNA expression in LAPC-4 cells. A: Western blot analysis of the effect of different concentrations of genistein on AR protein expression in LAPC-4 cells. B: Signal relative intensity was normalized to β-tubulin in LAPC-4 cells. C: Effects of increasing doses of genistein on the AR mRNA in LAPC-4 were measured by real-time RT-PCR. Results represent the means ± SD of three independent experiments. *p < 0.05, **p <0.01, for comparisons with the control groups.

**Figure 9:**
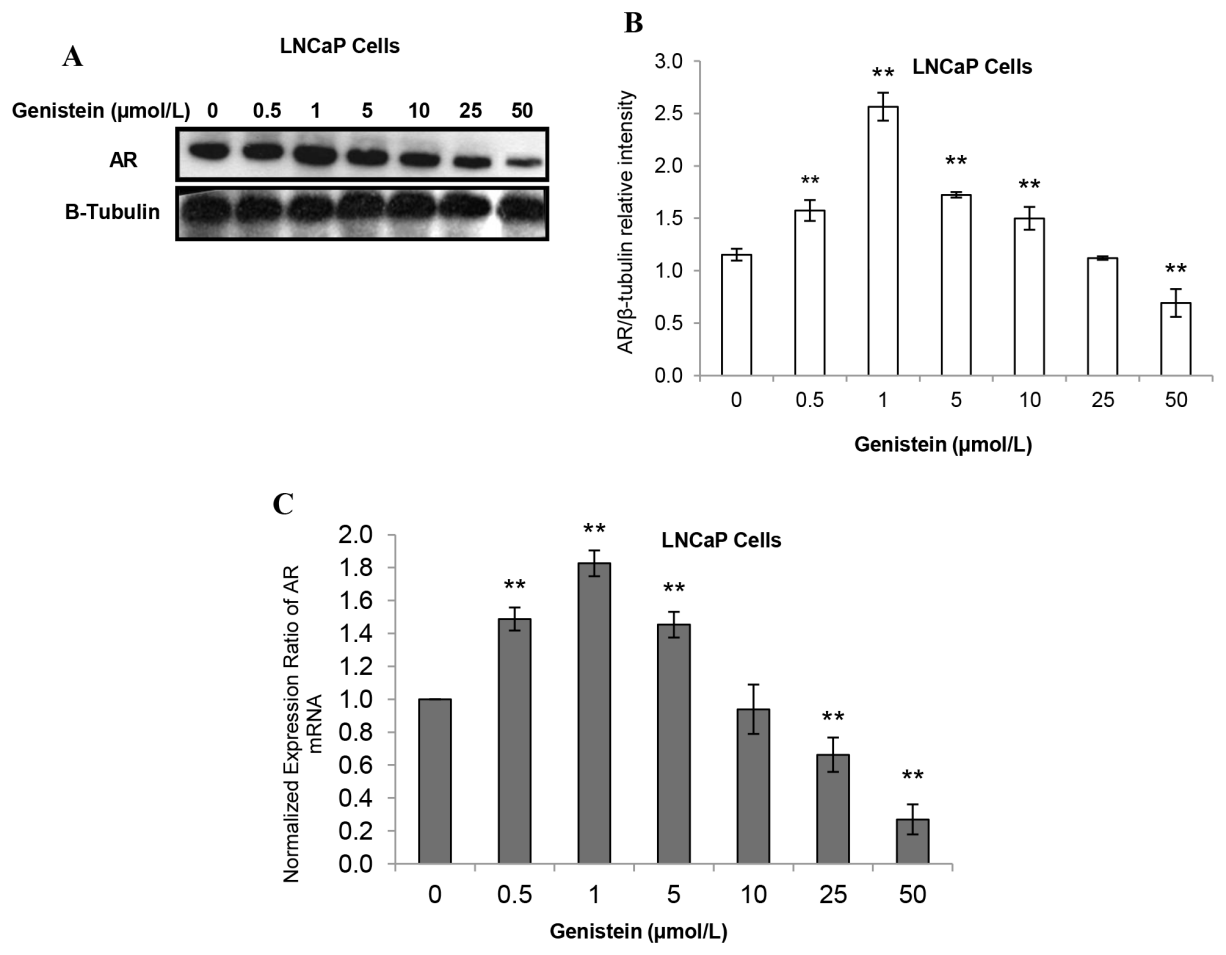
Genistein-mediated effects on AR protein and mRNA expression in LNCaP cells. A: Western blot analysis of the effect of different concentrations of genistein on AR protein expression in LNCaP cells. B: Signal relative intensity was normalized to β-tubulin in LNCaP cells. C: Effects of increasing doses of genistein on the AR mRNA in LNCaP were measured by real-time RT-PCR. Results represent the means ± SD of three independent experiments. *p < 0.05, **p <0.01, for comparisons with the control groups.

As a transcriptional factor, once bound to ligand, AR homodimerizes and translocates to the nucleus where it binds to androgen response elements for subsequent activation of transcription of targeted genes including those that control cell growth, as well as the AR gene itself (autoregulation) [59]. Since genistein was able to induce changes in the nuclear translocation of AR in both LAPC-4 and LNCaP cells, we investigated whether nuclear translocation of AR was associated with alterations in the mRNA and protein expression levels of AR in LAPC-4 and LNCaP cells treated with genistein. In LAPC-4 cells, AR protein and mRNA levels were decreased significantly (by 15% and 27%, respectively) in response to the lowest dose of genistein (0.5 µmol/L). This decline was dose-dependent, reaching 49% and 64% for AR protein (Figures 8A and 8B) and mRNA (Figure 8C), respectively, at a genistein dose of 25 µmol/L. In LNCaP cells, however, AR protein expression was increased by 16% in response to 0.5 µmol/L of genistein and peaked at 1µmol/L genistein (72% increase compared to the control). Above 10 µmol/L, genistein had inhibitory effects on AR protein expression in LNCaP cells (Figure 9A, 9B). Genistein-induced changes in AR mRNA levels in LNCaP cells mirrored the changes in AR protein expression (Figures 9C). These data demonstrate that low doses of genistein, in the absence of other steroids, are capable of inducing the expression of the T877A mutant AR through activating AR autoregulation.

### PSA Expression and Promoter Activity are Inhibited in LAPC-4 Cells, but Induced in LNCaP Cells in Response to Low Doses of Genistein

**Figure 10:**
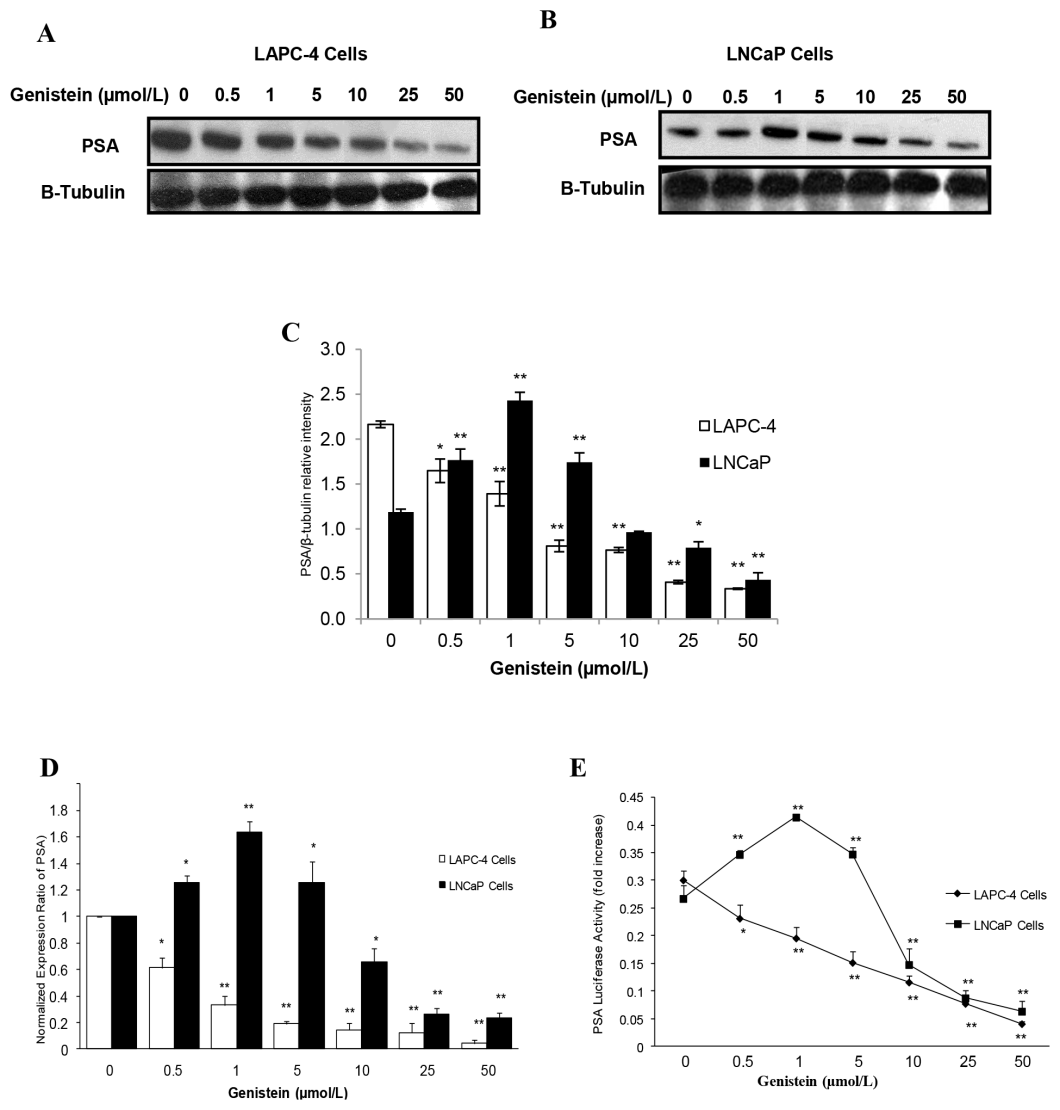
Effects of genistein on PSA expression and promoter activities in PCa cells. A and B: Western blot analysis of the effect of different concentrations of genistein on PSA protein expression in LAPC-4 and LNCaP cells, respectively. C: Signal relative intensity was normalized to β-tubulin. D: Quantitative assessment by real-time PCR of PSA mRNA expression in LAPC-4 and LNCaP cells in response to genistein treatment. E: Effects of genistein on PSA luciferase activity in LAPC-4 and LNCaP cells that were transfected with PSA promoter linked to a luciferase reporter in the pGL3-Basic vector. Results represent the means ± SD of three independent experiments. *p < 0.05, **p <0.01, for comparisons with the untreated control groups.

**Figure 11:**
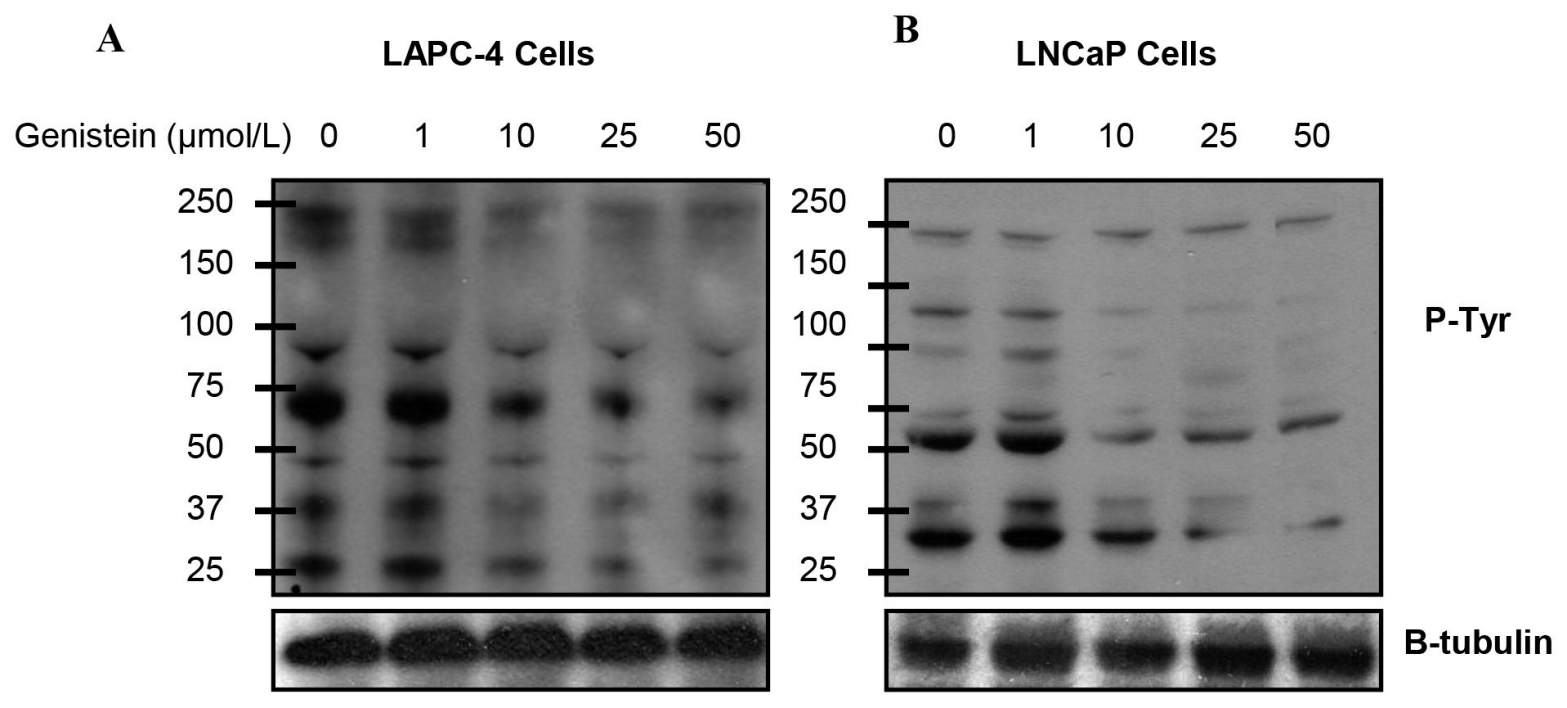
Effects of genistein on tyrosine kinase inhibition in PCa cells. Western blot analysis for the effect of genistein on tyrosine phosphorylation in a subset of proteins in LAPC-4(A) and LNCaP (B) cells using anti-phosphotyrosine antibody.

It is well documented that androgens stimulate PSA gene expression by up-regulating AR transcriptional activity [60]. To determine the effect of genistein on AR transcriptional activity, endogenous PSA protein and mRNA levels and PSA promoter activities were compared in LAPC-4 and LNCaP cells. Treatment with 1nmol/L R1881 strikingly increased PSA protein and mRNA levels in both cell lines compared to controls (data not shown). When cultured in steroid free media supplemented with genistein, LAPC-4 cells displayed a gradual dose-dependent reduction in PSA protein (Figure 10A and 10C) and mRNA (Figure 10D). In contrast, PSA protein and mRNA levels were increased in LNCaP cells at genistein concentrations between 0.5 and 5 µmol/L, resulting in 86%, and 63% increase, respectively. However, there was a significant reduction in PSA expression levels at genistein doses of 10 µmol/L or higher (Figure 10B, 10C and 10D). To determine whether the changes of PSA expression level corresponded to changes in PSA promoter activities, we measured PSA promoter luciferase activities in response to genistein treatment in both cell lines. A prominent dose-related decrease in PSA luciferase activity was found in LAPC-4 cells in response to all genistein concentrations tested (Figure 10E). In LNCaP cells, however, PSA promoter luciferase activities were increased by 29%, 55%, and 30% in response to 0.5, 1, and 5µmol/L genistein, respectively, and were reduced at genistein concentrations of 10µmol/L and greater (Figure 10E). In summary, these data indicate that low, physiological doses of genistein inhibited transcriptional activity of WT-AR in LAPC-4 cells, whereas it enhanced T877A mutant AR activation in LNCaP cells manifested by the induction of AR-regulated PSA gene. However, supraphysiological and pharmacological doses (≥10µmol/L) induced an inhibition of AR expression and transcriptional activity as well as cell proliferation.  In order to elucidate the mechanism behind this biphasic effect of genistein in LNCaP cells, we investigated the hypothesis that genistein, at higher doses, exerts inhibitory effects through a mechanism overrides its stimulatory effect on the mutant AR in LNCaP cells. Given the fact that the activity of the AR, as well as other cellular proteins fundamental for cell proliferation, is regulated by tyrosine phosphorylation, we sought to examine the effect of genistein on the state of tyrosine phosphorylation of cellular proteins by anti-phosphotyrosine immunoblotting. Our data demonstrated that genistein at doses of 10µmol/L or higher inhibited tyrosine kinase activity marked by suppression of tyrosine phosphorylation in a subset of cellular proteins in both LAPC-4 (Figure 11A) and LNCaP (Figure 11B) cell lines.

### Genistein Induces Mitogenesis in PCa Cells in the Presence of W741C and H874Y Mutant AR Proteins

**Figure 12:**
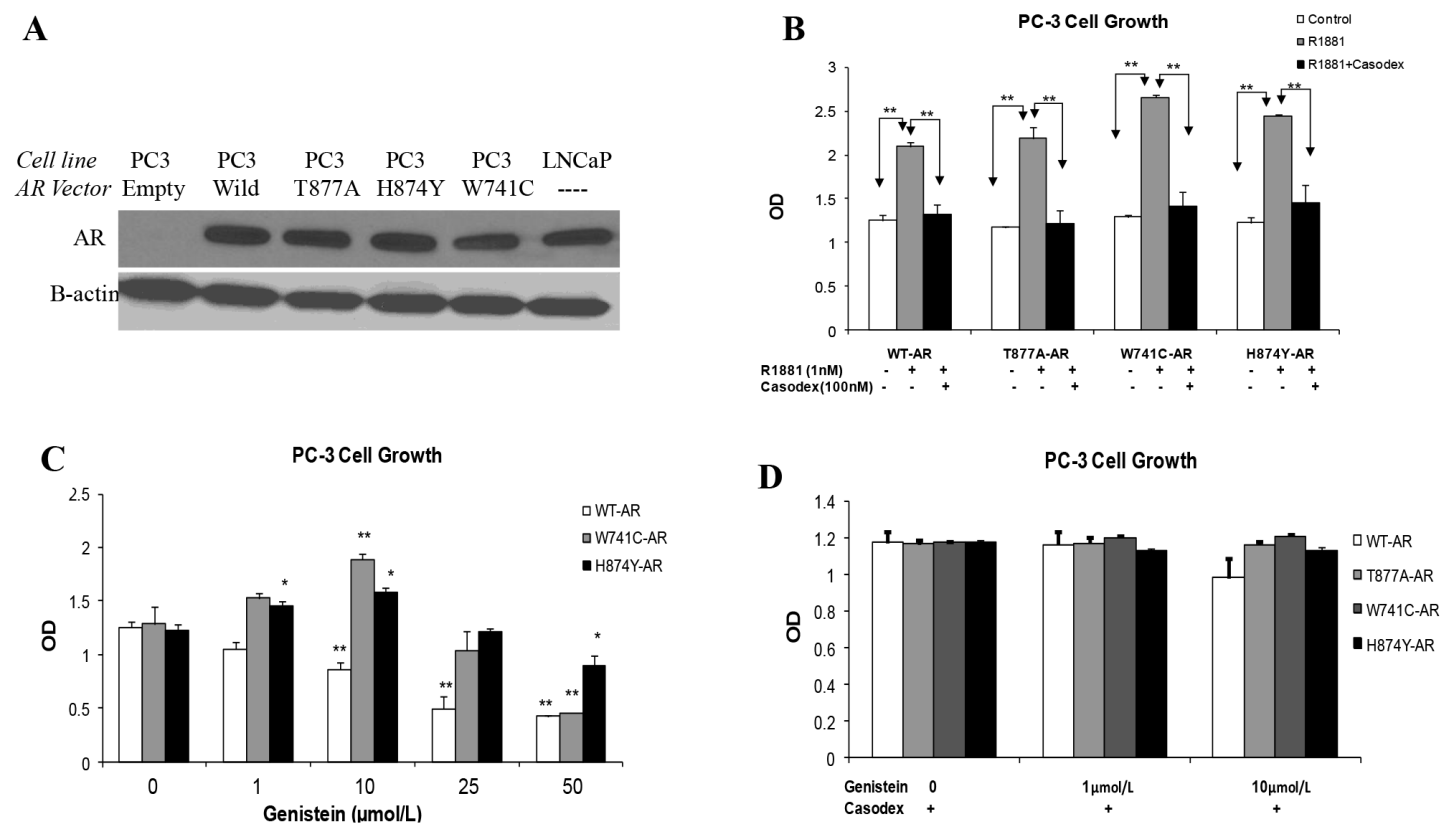
Effects of genistein on AR transfected PC-3 cell growth and viability measured by MTS assay. A: western blotting image for AR protein levels in null and transfected PC-3 cells relative to LNCaP cells. B: Growth of transfected PC-3 cell, with WT-AR or mutant ARs, after R1881 (1nmol/L) treatment with or without Casodex (100nmol/L). C: Effects of genistein treatment for 48 h on PC-3 cell growth (transfected with WT-AR or mutant AR, W741C and H874Y). D: The effect of combined treatment with Casodex (100nmol/L) and genistein for 48h on the proliferation of PC-3 transfected with WT-AR or mutant ARs.

We have shown that genistein, at physiological concentrations, enhances the proliferation of LNCaP cells that have T877A mutant AR. To explore whether genistein activity extends to other tumor-derived mutant ARs, we examined genistein effects on the proliferation of PC-3 cells we transiently transfected with two other AR mutants with promiscuous binding features (W741C and H874Y) [41,61]. A Western blot shows AR protein levels in transfected PC-3 cells relative to LNCaP cells is shown in Figure 12A. Transfected PC-3 cells were treated with vehicle, R1881 (1 nmol/L), or genistein for 48 hours. Synthetic androgen (R1881) treatment induced more marked proliferation in PC-3 cells transfected with mutant AR expression vector than PC-3 cells transfected with WT-AR (Figure 12B). While AR null PC-3 cells did not show any proliferation response to R1881 treatment. The proliferation of PC-3 cells transfected with these AR mutants was increased by low doses of genistein (≤ 10µmol/L), but higher doses (25 and 50 µmol/L) inhibited proliferation (Figure 12C). This biphasic response was remarkably similar to that displayed by LNCaP cells or PC-3 cells upon transfection with T877A mutant AR (Figures 1B and 1E). Ten µmol/L of genistein stimulated cell proliferation in PC-3 cells transfected with the W741C mutant AR by 50% relative to untreated controls, and by 20% in cells transfected with the W741C AR. To verify that genistein action in PC-3 cells is dependent on mutant AR activity; experiments were repeated in the presence of Casodex which abolished genistein-induced cell proliferation in PC-3 cells transfected with H874Y or W741C mutants (Figure 12D), confirming that this effect is dependent on the activation of the mutant AR. 

## Discussion

This study addressed two fundamental questions: First, whether genistein in its physiological concentrations can serve as a potential androgen-independent stimulator of the promiscuously mutant AR present in patients with advanced PCa, which might lead to therapeutic relapse in patients who receive androgen ablation therapy. Second, whether physiological concentrations of genistein exert different effects on PCa cells that have wild type AR than cells with mutant AR. Accordingly, this study may offer a more understanding of the expected beneficial Vs harmful effects of genistein in different stages of PCa without creation of a misconception about the hazardous effects of genistein. 

We showed that genistein action on PCa cells is dose-dependent and that the biological outcome of genistein exposure depends on the mutational status of AR. Genistein inhibited cell proliferation and induced apoptosis in LAPC-4 PCa cells that have WT-AR in a linear dose-dependent fashion. In contrast, the response of LNCaP cells to genistein was biphasic with stimulation of cell proliferation at physiological concentrations (less than 10 μmol/L) and inhibitory responses at doses of 25 μmol/L and above. These stimulatory effects in LNCaP cells at low genistein concentrations were abolished by blocking the AR using the AR antagonist, Casodex, indicating that the stimulatory action by genistein in LNCaP cells were most probably due to AR activation and related to the presence of the T877A mutation in the AR of this cell line. Thus, the mutational status of AR appears to influence the bioactivity of genistein in PCa cells. The ability of genistein to activate the mutant AR, but not the WT-AR, was confirmed by using AR-negative PC-3 cells transfected with either WT-AR or T877A mutant AR; an approach that avoids potential biological differences between different cell lines when using LNCaP and LAPC-4 cells. Genistein, at physiological achievable concentrations, had significant stimulatory effects on growth of PC-3 cells expressing the T877A-AR and inhibitory effects on proliferation of PC-3 cells transiently transfected with the WT-AR. The differences we observed in response to genistein among cells that have WT-AR and cells with the mutant AR may explain apparent discrepancies reported in the literature. Similar biphasic proliferative effects of genistein on LNCaP cells have also been observed by Maggiolini et al. [45] who attributed this effect to the presence of the T877A mutant AR. Our observation of the absence of such a biphasic genistein effect on growth of PCa cells harboring a wild type AR provides novel direct evidence for this notion. It has been reported that genistein exerts a tyrosine kinase inhibitory effect in PCa cells, which results in inhibition of tyrosine phosphorylation and eventually the activity of several growth factors and proteins essential for promoting cell proliferation in PCa cells including AR. In our data, we confirmed the tyrosine kinase inhibitory properties of genistein at doses of 10µmol/L or more in both cell lines, which provides an explanation to the biphasic effect observed in LNCaP cells. 

The ability of physiological concentrations of genistein to activate tumor derived AR was considerably conserved across other mutant ARs. In this study, we investigated genistein action on two other tumor-derived AR proteins; W741C-AR and H874Y-AR that were identified in patients with metastatic androgen-independent disease following androgen ablation therapy and have been shown to respond to various non-androgenic steroids [39,40]. Low doses of genistein (≤ 10μmol/L) enhanced the proliferation of PC-3 cells transfected with these tumor-derived AR mutant proteins, but not the WT- AR, and this effect was abrogated by Casodex. Perhaps due to a relatively low abundance in transiently transfected PC-3 cells of these two mutant ARs, higher concentrations (10 µmol/L) were required to achieve maximum enhancement of cell proliferation in this system relative to LNCaP cells. 

Interestingly, the ability of genistein to inhibit proliferation of the androgen-independent cell line PC-3 was modest by comparison with PCa cells that are dependent on androgen for growth such as LNCaP and LAPC-4. Strikingly, the anti-proliferative effect of genistein on PC-3 cells was amplified when PC-3 cells were transfected with WT-AR and diminished when the WT-AR was blocked with Casodex. Thus, the anti-mitogenic action of genistein correlates with the presence of functional AR in PCa cells and is associated with AR activation, but an AR-independent mechanism also seem to be involved. 

Several recent studies have shown that genistein inhibits PCa cell proliferation at high micromolar concentrations [62–64]. We also observed an anti-proliferative effect of genistein at concentrations of 25 µmol/L and higher, both in cells that contain WT-AR and cells with mutant ARs. However, this outcome of genistein action holds limited biological relevance because these concentrations do not reflect the physiological achievable doses in human prostate tissue [7,8,65]. We observed that genistein at low concentrations stimulated proliferation of PCa cells that express promiscuously mutant ARs (AR-T877A, W741C and H874Y). Thus, our findings suggest that the effect of genistein on PCa cell proliferation can vary dramatically depend on both the doses tested and the mutational status of the AR.

In line with the ability of genistein to enhance the AR activity in PCa cells with the T877A mutation, *in silico* modeling predicted that genistein docks with the T877A-AR more efficiently with almost 2 folds higher free energy of binding than with the WT-AR. These observations were further strengthened by our data from the ^3^[H]-R1881competitive binding assay showing that genistein, starting at low physiological doses (1µmol/L), effectively competed with labeled androgen for binding to both the WT and mutant AR in LAPC-4 and LNCaP cells, respectively. Collectively, these data indicate that genistein binds to the AR, but with more affinity and stability to the T877A mutant AR than to the WT AR. 

AR, a member of the nuclear hormone receptor family, is activated by binding to its specific ligand, dihydrotestosterone, or is constitutively activated [66–69]. We speculate that the ability of genistein, demonstrated in our data, to interact physically with the LBD of AR might be a possible mechanism that results in alteration of AR nuclear localization in PCa cells. The recent exceptional work of Zhou et al [70] has successfully simulated the impact of the T877A mutation on the ligand-induced rearrangement of helix-12 at the AR LBD, which plays a critical role in AR transactivation. They demonstrated that helix-12 rearranges into the agonist conformation in the T877A mutant receptor, but into the antagonist conformations in the WT receptor in response to the AR antagonist; hydroxyflutamide. These findings may provide a plausible explanation for the genistein mediated enhancement of proliferation in LNCaP cells that have T877A mutant AR, while exerting suppressing effects in LAPC-4 cells with WT-AR. One possible scenario is that genistein serves as an antagonist for the WT-AR and shifts to an agonist in the presence of the T877A mutant AR, similar to what has been demonstrated for the AR antagonist, Flutamide [70,71]. This hypothesis is strengthened by our data showing that genistein effectively inhibited AR nuclear localization in LAPC-4 cells. However, it induced AR translocation in LNCaP cells significantly. 

Ultimately, assessment of the status of AR activity is critical in elucidating the biological impact of this potential AR binding of genistein in both LNCaP and LAPC-4 cells. Thus, we examined the effect of a range of genistein doses on AR transcriptional activity in both cell lines, using expression of the AR-responsive gene PSA as a surrogate marker. Given that AR possesses an autoregulatory function, we employed the amount of AR expression as another indicator of AR activation. Suppression of LAPC-4 cell proliferation was tightly associated with a reduction of AR protein and mRNA levels, as well as PSA expression in response to increasing genistein from physiological to supraphysiological doses. Similarly, in LNCaP cells, the biphasic proliferation response was associated with a parallel biphasic response of AR and PSA expression. Collectively, our data provide strong evidence that the differential response of LNCaP and LAPC-4 cells to genistein is AR mediated and T877A mutation dependent. 

Results from other *in vitro* studies examining the effects of genistein on PCa cells have yielded conflicting results; some studies demonstrated that genistein downregulates AR expression and inhibits cell proliferation [24,26,27], whereas others reported stimulatory effects of genistein on PCa cell proliferation and AR activity [45–47]. Our findings indicate that these discrepancies are conceivably attributable to (a) the utilization of the LNCaP PCa cell line because it contains the T877A AR mutation and (b) the limited range of mostly pharmacological genistein concentrations used in most of these studies. 

Although *in vitro* studies with purified soy compounds in cancer cells may not reflect effects of dietary soy on prostate cancer *in vivo*, epidemiological observations suggest a protective role of soy in PCa [4,5], implicating genistein because it is the most abundant and biologically active soy isoflavone. We observed maximal stimulation of cell proliferation and inhibition of apoptosis in response to genistein in LNCaP cells at a dose of 1µmol/L, indicating that apparently modest exposure to the phytoestrogen genistein can have significant adverse biological consequences for prostate cancers that carry promiscuously mutant AR. The concentrations we used are well within the range of physiological levels that can be achieved by humans through daily soy consumption. The upper limit of genistein serum levels in Japanese people consuming soy-rich diets is in the order of 2 µmol/L [7,8]. Levels of soy isoflavones in prostate tissue may exceed that in serum by 4 to 6-fold [65], indicating that genistein levels in prostate tissue do not exceed 8 to 12 µmol/L, emphasizing the importance of the physiological concentrations used in our study. 

Cumulatively, our data add a new insight to the understanding of the bioactivities of genistein in PCa, which are affected by several factors including the dose of genistein and the mutational status of the AR. This study indicated that genistein at low, physiologically relevant exposure levels can be an endocrine disruptor and can result in altered activity of multiple tumor-derived mutant AR alleles. Therefore, genistein, especially at lower doses, can function as a potential ‘‘hormone sensitizer’’ of the mutant ARs present in advanced PCa following androgen ablation therapy, thereby possibly contributing toward therapeutic relapse in soy consuming PCa patients who have cancers carrying the T877A or similar mutations such as W741C and H874Y. The T877A AR variant is of obvious biological relevance as it has been reported in up to 12.5% of hormone-refractory PCa [40,72,73] and is considered an example of a range of AR variants in PCa that have promiscuous ligand specificity, such as the K580R and the V715M variants that are induced by estradiol at 0.1nmol/L [39,48,74]. 

Investigations with the orthotopic xenograft models using human PCa cell lines with these AR-LBD mutations are warranted in order to validate our findings, given the close resemblance of these models to the actual prostate tissue conditions of stromal cells and physiological and hormonal milieu. Furthermore, it is essential to investigate whether other soy phytoestrogens such as daidzein, quercetin, and equol have similar effects. It will be also of interest to assess contributions of AR cofactors to genistein-mediated transactivation of mutant ARs. In fact, it has been reported that certain cofactors can enhance AR transactivation in the presence of non-androgenic steroids and that deregulation of these cofactors had been implicated in prostate cancer evolution to androgen independence [75–77]. 
